# Sociodemographic Differences in Pain Medication Usage and Healthcare Provider Utilization Among Adults With Chronic Low Back Pain

**DOI:** 10.3389/fpain.2021.806310

**Published:** 2022-01-26

**Authors:** Kristen Allen-Watts, Andrew M. Sims, Taylor L. Buchanan, Danica J. B. DeJesus, Tammie L. Quinn, Thomas W. Buford, Burel R. Goodin, Deanna D. Rumble

**Affiliations:** ^1^Department of Biostatistics, University of Alabama at Birmingham, Birmingham, AL, United States; ^2^School of Medicine, University of Alabama at Birmingham, Birmingham, AL, United States; ^3^Department of Psychology, University of Alabama at Birmingham, Birmingham, AL, United States; ^4^Geriatric Research Education and Clinical Center, Birmingham Veterans Affairs (VA) Medical Center, Birmingham, AL, United States; ^5^Department of Psychology and Counseling, University of Central Arkansas, Conway, AR, United States

**Keywords:** chronic low back pain (cLBP), disparities, provider utilization, pain treatment, access to healthcare

## Abstract

Chronic low back pain (cLBP) is the most common reason for individual suffering and health care utilization in adults. Ample evidence suggests sociodemographic variables and socioeconomic status (SES) influence pain. However, a framework informing associations on race, SES, and the utilization of pharmacologic therapies and provider type are limited—particularly in cLBP. Thus, this study examined the extent to which sociodemographic (i.e., age, race, and gender) and socioeconomic factors (i.e., national area deprivation index, NADI) influence pain treatment (i.e., NSAIDs, opioids, antidepressants, and non-NSAIDs) and provider utilization for cLBP (i.e., no provider care, primary care, or tertiary care). Eligible participants with cLBP completed a series of questionnaires. Of the 174 participants, 58% were women, 59% were non-Hispanic Black (NHB), and the mean age was 46.10 (SD 13.58). Based on NADI distributions by race, NHB participants lived in more socioeconomically disadvantaged neighborhoods (*p* < 0.001) than non-Hispanic White (NHW) adults. Results suggested that the use of one or more pharmacologic therapies was associated with race (*p* = 0.021). Specifically, NHW adults were two times more likely to take one or more pharmacologic therapies than NHBs (*p* = 0.009). NHWs were also more likely to use NSAIDs (*p* = 0.041) and antidepressants (*p* < 0.001) than NHBs. Furthermore, provider utilization was significantly associated with gender (*p* = 0.037) and age (*p* = 0.018); which suggests older women were more likely to use primary or tertiary care. Findings from this study expand on the existing literature as it relates to associations between disparities in access to healthcare providers and access to medications. Future research should seek to understand differences in age and utilization of primary or tertiary care providers and continue to examine the influence of sociodemographic and SES factors to cLBP and compare with other types of chronic pain.

## Introduction

Chronic pain is a public health problem that imposes a significant social and economic burden and reduces the quality of life and well-being for adults worldwide (1–7). Chronic low back pain (cLBP) refers to a persistent or recurring pain in the lumbar region of the spine to the pelvis (8) and is the most common reason for individual suffering and utilization of health care services across the lifespan (3). The experience of pain can be influenced by several factors, including, but not limited to, race, socioeconomic status (SES) and neighborhood-level characteristics, advanced age, and gender (9, 10). Mounting evidence suggests disparities and inequities in pain management exist among vulnerable subgroups that are influenced by social determinants of health (5, 7). Specifically, chronic pain is often under-assessed, under-diagnosed, and under-treated in non-Hispanic Black (NHB) adults (11) and NHBs are more likely to have their pain discounted and less likely to be screened for pain than their non-Hispanic White (NHW) counterparts due to systematic factors and implicit bias in the healthcare setting (7). Furthermore, adults with cLBP from low SES experience significant financial burden, decreased quality of life from chronic pain symptoms, and greater morbidity and mortality than individuals at higher SES levels (12–14).

cLBP has a complex etiology (15) that impacts physical and emotional function (16) and requires a varied approach to treatment and management strategies (17). Patients often seek primary care providers (PCP) or specialized care from tertiary providers for pain management (18, 19). Although the literature is mixed on the efficacy of treatment in primary care vs. tertiary care settings, most patients utilize PCP due to accessibility and established relationships between the patient and the provider (19). Regardless of care setting, cLBP is a dynamic, multidimensional, comorbid condition that requires a tailored approach to evaluating the risks and side effects of pain management (19), which is a key component for effective pharmacologic therapies regarding cLBP diagnosis (20). The most commonly prescribed pharmacologic therapies, include antidepressants (21), non-steroidal anti-inflammatory drugs (NSAIDs), (22) opioids, and non-NSAID medications such as acetaminophen (23).

Disparities in pain treatment and management strategies are well-known (24); however, a framework informing associations on race, SES, type of provider seen and use of pharmacologic therapies, specifically in cLBP, is lacking. Thus, the purpose of this study was to examine whether sociodemographic factors (i.e., age, race, and gender) and socioeconomic factors (i.e., national area deprivation index, NADI) influence pain treatment (i.e., NSAIDs, opioids, antidepressants, and non-NSAIDs) and provider utilization for cLBP (i.e., no provider care, primary care, or tertiary care).

## Materials and Methods

### Study Design and Setting

**Figure 1:**
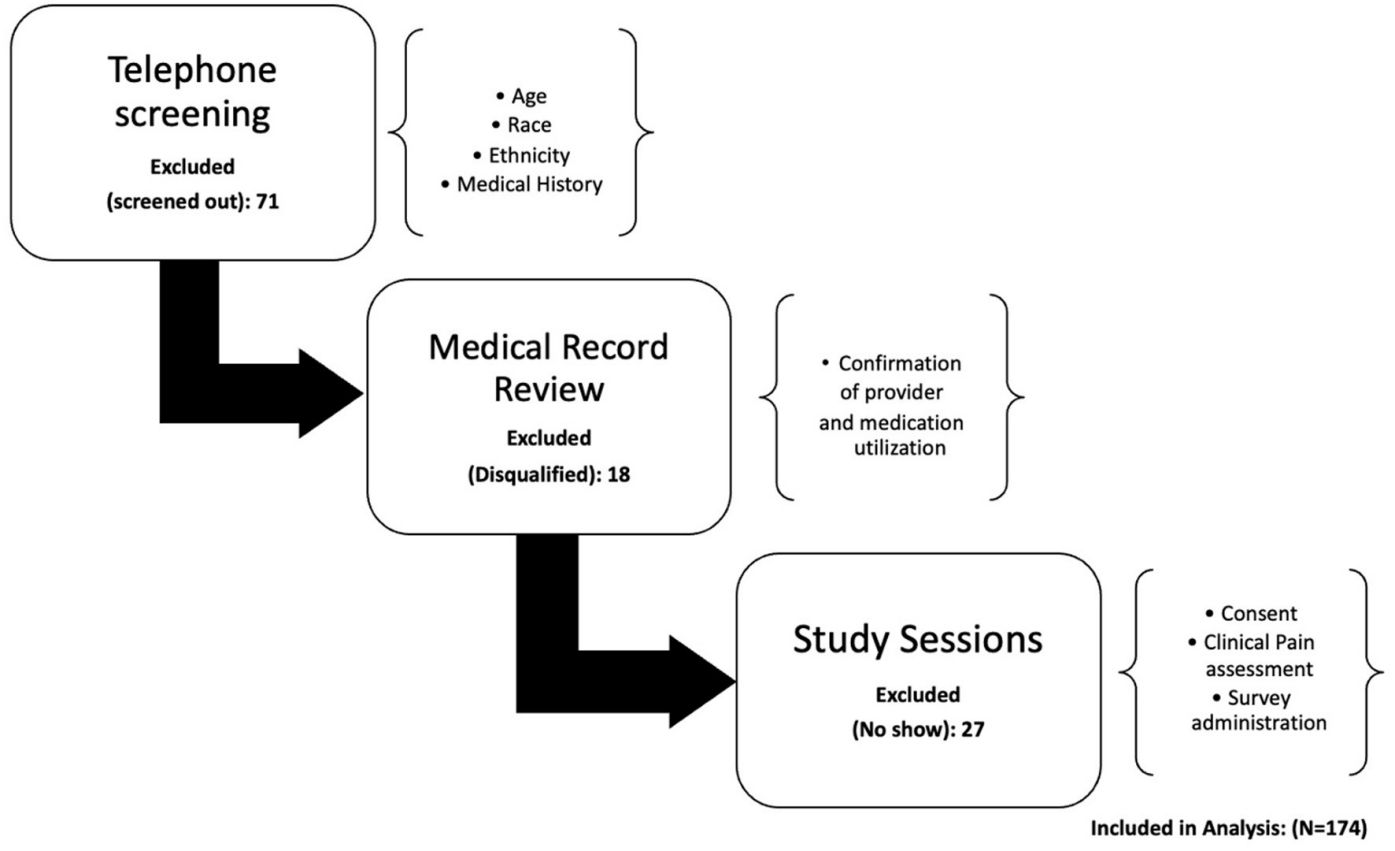
Flow chart.

This study is part of a larger ongoing investigation examining ethnic/racial differences in cLBP severity and disability referred to as the Examining Racial and SocioEconomic Disparities (ERASED) in Chronic Low Back Pain Study (NIH grant# R01MD010441). The current study employed a secondary analysis of data to characterize the impact of sociodemographic variables associated with pharmacologic approaches and provider utilization in participants recruited from April 2017-August 2021. Details of the full protocol of ERASED have been described in detail elsewhere (25, 26). Briefly, ERASED uses the theory of fundamental causes (27) as a socioeconomic framework to explain why cLBP severity, disability, and factors that predict these outcomes may differ based on race and socioeconomic status (SES) (25, 26). For the parent study, a recruitment flier was developed and posted at an academically affiliated pain treatment clinic and the surrounding academic community. Study staff screened and determined eligibility using the potential study participant's electronic medical record. Participants were eligible if they were: (1) English speaking; (2) 18–85 years old; (3) identified as either NHB or NHW, (4) had low back pain for at least six consecutive months, and cLBP was primary pain experience; (5) no surgeries or lawsuits related to cLBP (8). A comprehensive list of exclusion criteria has been previously reported elsewhere (25, 28). Briefly, participants were ineligible if they had any concurrent medical conditions or coexisting diseases. The parent study is comprised of two laboratory-based sessions (Figure 1). In session one, resting blood pressure, body mass index (BMI), literacy evaluation, and measures related to SES and clinical pain assessment (CPA) were collected in addition to an endogenous pain modulation. Approximately 1 week later, participants returned for a second session to complete biomarker and functional performance measures. During this session, additional questionnaires were collected, and participants were asked to report any changes in quality of cLBP since session one. All participants were then contacted by phone once a week for 4 weeks following session two to report on symptoms of pain, mood, sleep, disability, and interference of pain with daily activities. For the current study, the measures and procedures were limited to those who completed measures regarding sociodemographics, pain characteristics, and pain care utilization. Patients reported medication usage in the screening form, CPA questionnaire, and verified using the patients' medical records. Patients provided information on provider visits in the CPA questionnaire and verified using medical records. The ERASED study was conducted in accordance with the Research Task Force of the NIH Pain Consortiums' cLBP research standards (8) and all procedures conformed to the standards set by the latest revision of the Declaration of Helsinki and reviewed and approved by the Institutional Review Board at the University of Alabama at Birmingham (UAB) (IRB-170119003).

### Measures

#### Sociodemographic Characteristics

Sociodemographic information including race/ethnicity, age, and gender were collected from all participants. For inclusion into this study, participants had to self-identify as either NHB or NHW. Gender was reported as either man, woman, transgender man, transgender woman, or other (e.g., gender fluid). Furthermore, each participant reported their address and zip code, which was used for the calculating their National Area Deprivation Index (NADI) (29). The NADI considers seventeen SES variables related to education, income, employment, and housing to assign the participants a 1–100 value based on their 9-digit zip code with higher scores indicating greater levels of neighborhood deprivation (29).

#### cLBP History

Information was provided regarding presence and duration of cLBP to ensure that participants met study inclusion criteria. This history also asked participants to document their current medication list, including analgesic medications taken specifically for cLBP (i.e., NSAIDs, opioids, antidepressants, and non-NSAIDs). Participants reported their most recent patterns of provider utilization for cLBP treatment. Provider information included type of provider seen for cLBP treatment [i.e., no provider care, primary care, or tertiary care (e.g., pain medicine/anesthesiology, physical therapy)], frequency of provider visits, and provider satisfaction ratings.

#### Current Pain Severity

The Brief Pain Inventory (BPI-SF) short form (30), was used to assess participants' self-reported pain severity (30). Pain severity was calculated by averaging the pain severity items that reflect pain at its worst in the last 24 h, pain at its least in the last 24 h, average pain, and pain right now (i.e., current pain). Higher scores are indicative of greater cLBP severity (30).

### Statistical Analyses

Data were analyzed using SPSS version 28.0. Descriptive statistics were computed for the cLBP group. All data are presented as percentages or as means and standard deviations (SD). The dependent variables included in the analyses were the use of one or more pharmacologic therapies, dichotomized as use of opioids, antidepressants, NSAIDs and non-NSAIDs, or no use. Provider type was classified as no care, primary care, or tertiary care. This recoded variable was categorized as an ordinal measure with no provider serving as the lowest level of care, primary provider as the middle level of care, and tertiary serving as the highest level of care. Because various studies suggest demographic factors and socioeconomic status and neighborhood deprivation contribute to the pain experience (20, 26), we included race, gender, age, BPI (pain severity), and NADI as our covariates. Chi-square was used to test for associations between race, gender, utilization of provider type and use of opioids, NSAIDs, antidepressants, and non-NSAIDs. One-way ANOVA was applied to examine whether age significantly differed according to type of provider utilized and use of one or more pharmacologic therapies. Independent *t*-tests were used to examine differences by race and gender. Spearman correlations were used to examine relationships among continuously measured variables such as BPI-SF and NADI. To examine whether covariates predict the use of provider type, we applied a proportional odds model and used the test of parallel lines to test the proportional odds assumption. A logistic regression model was applied to predict the use of one or more pharmacologic therapies on the selected sociodemographic and SES factors. Approximately 10% of the data was excluded in the final analysis due to missing values.

## Results

### Participant Characteristics

The descriptive statistics and participant characteristics are displayed in Table 1. Of the 174 participants, 58% were women (42% men); nobody identified as transgender or other. Further, 59% were NHB and the remaining 41% were NHW. The mean age was 46.10 years (SD 13.58). Our analysis revealed 50% of women used a primary care provider for cLBP compared to 42% of men and 33% of women used a tertiary care provider for cLBP compared to 26% of men. Men were more likely to report the use of no primary care provider for cLBP than women (33% compared to 18%, respectively). Regarding the use of one or more pharmacologic therapies, 88% of participants stated they were currently taking at least one pharmacologic agent for cLBP management. Thirty one percent of NHWs used antidepressants compared to 8% of NHBs and 60% of NHWs used NSAIDs compared to 44% of NHBs. An independent *t*-test was conducted to compare NADI by race [NHB *M* = 69.09 (SD = 27.93); NHW *M* = 33.66 (SD = 52.01), *t* = 5.090, *p* < 0.001]. Based on these results, NHB participants had significantly greater NADI than NHW participants, such that NHB participants tended to live in more socioeconomically disadvantaged neighborhoods. Results further revealed a significant race difference in pain severity reported on the BPI-SF, such that NHB participants [*M* = 5.31 (SD = 2.05)] reported greater pain severity than NHW participants [*M* = 4.28 (SD = 1.70)] (*t* = 3.46, *p* < 0.001). Greater neighborhood disadvantage represented by higher NADI was significantly associated with greater pain severity (r_spearman_ = 0.329, *p* < 0.001). The pain severity reported by men [*M* = 5.00 (SD = 1.99)] and women [*M* = 4.86 (SD = 2.00)] did not significantly differ (*t* = 0.469, *p* = 0.320), and age was not significantly associated with pain severity (r_spearman_ = 0.068; *p* = 0.185).

**Table 1:** Descriptive characteristics, provider utilization, and use of pharmacologic therapies information for participants (*n* = 174).

**Demographic characteristics**	**Mean (SD) or %**
Age (years)	46.10 (13.58)
**Gender (% female)**	
(% women)	58.0%
(% men)	42.0%
**Race (% NH black)**	
(% non-hispanic black)	59.3%
(% non-hispanic white)	40.7%
National area deprivation index (NADI)	54.01 (43.45)
BPI pain severity	4.92 (2.00)
**Provider utilization characteristics**	
No provider care	23.3%
Primary care	46.6%
Tertiary care	30.1%
**Pharmacologic therapies characteristics**	
No pharmacologic therapies	39.7%
One or more pharmacologic therapies	60.3%

### Chi-Square Models of Race and Gender Associations

Based on our results, regarding the use of pharmacologic therapies, there was a significant association between race and the use of NSAIDs and antidepressants. Specifically, NHW adults were more likely to use NSAIDs [$\chi_{\left(1\right)}^{2}$ = 4.189, *p* = 0.041] and antidepressants [$\chi_{\left(1\right)}^{2}$ = 16.036, *p* < 0.001]. There were no significant associations between race and the use of opioids [$\chi_{\left(1\right)}^{2}$ = 0.018, *p* = 0.895] or non-NSAIDs [$\chi_{\left(1\right)}^{2}$ = 0.132, *p* = 0.716]. Additionally, gender was not associated with the use of opioids [$\chi_{\left(1\right)}^{2}$ = 0.000, *p* = 0.989], NSAIDs [$\chi_{\left(1\right)}^{2}$ = 0.590, *p* = 0.442], antidepressants [$\chi_{\left(1\right)}^{2}$ = 2.127, *p* = 0.145], or non-NSAIDs [$\chi_{\left(1\right)}^{2}$ = 0.046, *p* = 0.831]. Lastly, race was not associated with type of provider utilized [$\chi_{\left(2\right)}^{2}$ = 3.660, *p* = 0.160] nor gender with type of provider utilized [$\chi_{\left(2\right)}^{2}$ = 4.447, *p* = 0.108].

### Pharmacologic Therapy Utilization

Using a binary logistic regression model, our findings suggest a statistically significant association between race and use of one or more pharmacologic therapies compared to none. We found that NHW adults were two times more likely to take one or more pharmacologic therapies than NHBs (*p* = 0.013; OR = 2.67; CI: 1.23, 5.79). This relationship held even after adjusting for other covariates. The OR indicates that NHWs have 167% higher odds of using one or more pharmacologic therapies than NHBs. Use of one or more pharmacologic therapies was not significantly associated with age (*p* = 0.217; OR: 1.02; CI: 0.990, 1.044), gender (*p* = 0.113; OR: 1.77; CI: 0.874, 3.57), pain severity (*p* = 0.233; OR: 1.12; CI: 0.932, 1.34) or NADI (*p* = 0.424; OR: 0.996; CI: 0.987, 1.006). Results are shown in Table 2.

**Table 2:** Sociodemographic and SES predictors of use of one or more pharmacologic therapies (*n* = 174).

**Variable**	**Odds ratio**	CI^*^
Age (years)	1.02	(0.990, 1.044)
Gender	1.77	(0.874, 3.57)
Race^*^	2.67	(1.23, 5.79)
National area deprivation index (NADI)	1.00	(0.987, 1.006)
BPI pain severity	1.12	(0.932, 1.34)

* *significant predictors*.

### Provider Utilization

The proportional odds model indicated a significant improvement in fit of the final model (*p* = 0.046). Examination of the proportional odds assumption showed no evidence of a violation of model assumptions (*p* = 0.187). Based on the regression coefficients and significance test for each of the covariates in the model, women were more likely to utilize primary or tertiary care (*p* = 0.041; OR = 2.09; CI: 1.031, 4.228). Furthermore, older participants were more likely to utilize primary or tertiary care than younger participants (*p* = 0.019). These findings suggest women have 2 times greater odds of utilizing a primary or tertiary care compared to men, and for every 10 years increase in age is associated with 30% increased odds of utilizing primary or tertiary care (OR: 1.030; CI: 1.005, 1.056). Race (*p* = 0.229; OR = 1.57; CI: 0.754, 3.248), pain severity (*p* = 0.959; OR: 1.005; CI: 0.841, 1.200) and NADI (*p* = 0.440; OR = 0.97; CI: 0.988, 1.005) were not significant predictors of provider utilization in the model shown in Table 3.

**Table 3:** Sociodemographic and SES predictors of provider utilization (*n* = 174).

**Variable**	**Odds ratio**	CI^*^
Age (10 years)^*^	1.03	(1.005, 1.056)
Gender^*^	2.09	(1.031, 4.228)
Race	1.57	(0.754, 3.248)
National area deprivation index (NADI)	0.97	(0.988, 1.005)
BPI pain severity	1.005	(0.841, 1.200)

* *significant predictors*.

## Discussion

Our study intended to determine differences in pain treatment and provider utilization among adults with cLBP. Specifically, we focused our efforts on examining differences in selected sociodemographic factors (i.e., race, age, gender) and SES (i.e., NADI) and the use of one or more pharmacologic therapies and provider type (i.e., no provider care, primary care, or tertiary care). Based on results from our study, two primary findings emerged. Firstly, NHW participants with cLBP were two times more likely to take one or more pharmacologic therapies compared to NHBs. Specifically, our findings suggest that NHWs are more likely to take NSAIDs and antidepressants than NHBs. These results are consistent with the literature as it relates to SES and sociodemographic factors influencing physician prescribing behaviors (31–33). However, on the patient end of the spectrum, socioeconomic factors may also influence the ability to afford certain medications or obtain prescriptions (34). For the current study, the NADI for NHW participants was lower than that of the NHB participants. This indicates that NHW participants live in areas that have greater socioeconomic advantages in variables such as education, income, employment, and housing (29), and these factors may increase the ability to access and afford healthcare as well as impact the ability to adhere to medication, even if available (35). Moreover, studies suggest differences in attitudes and preferences for treatment among NHWs and NHBs. For example, research has shown that NHBs are less likely to seek mental help related to depressive symptoms due to the cultural stigma surrounding mental health in the NHB community (36). This may explain why even though antidepressants have been proven as an effective intervention for pain-relief in cLBP patients (37), use of the pharmacologic agent is either under-utilized or under-reported in the NHB community (21, 32). Stigma related to medication use is particularly salient for opioid use. Although race was not a significant predictor for the use of opioids in the current study, cultural and anti-opioid stigma may play a role in participants choosing to under-report (38). Due to the opioid crisis, anti-opioid campaigns to reduce opioid stigma are widely documented within the literature (39–41). However, negative attitudes, perceptions, and stereotypes about people who use opioids persist and may hinder patient disclosure of opioid use. Patient non-disclosure is also the case for the use of non-prescription opioids, which are commonly obtained through family and friends, and may involve non-standard behaviors such as crushing, snorting, or chewing (42). While these non-prescription opioids and administration methods could potentially be used for pain management, this type of usage is often classified as opioid abuse or recreational use and thus, unlikely to be reported.

Another key finding in the current study is the influence of sociodemographic factors, such as age, and the utilization of health care services, specifically, use of primary or tertiary provider care in cLBP. A well-documented explanation for this finding is that older adults tend to be enrolled in Medicare by age 65, and thus have increased access to health providers compared to younger adults in low socioeconomic or under-resourced areas who may not have access to primary or specialty care services (43). Additionally, older adults often utilize providers because they tend to be at greater risk for multiple comorbidities and decline in physical function than younger adults (44–46). The current study also suggest that women are more likely to utilize primary or tertiary care for cLBP than men, which is consistent with previous literature which posits men are less likely to utilize health care services and more likely to adopt negative health related behaviors, such as ignoring pain and other health-related symptoms than women (47, 48). Our findings suggest, gender did not significantly differ according to pain severity. However, consistent with the literature, there were significant differences in pain severity in race and NADI. Specifically, NHB participants reported greater pain severity than NHWs and greater neighborhood deprivation was associated with greater pain severity (28).

There were several limitations of the current study. Firstly, this study was cross-sectional and cross-sectional studies do not describe cause and effect relationships; therefore, causal associations cannot be made. Additionally, older participants may have difficulty recalling past events, especially use of pharmacologic therapies, which may have biased their self-report and thus the results. Furthermore, the use of socially acceptable responses is also a limitation. Social desirability bias occurs when participants respond to questions (or report on a behavior) in a manner that is favorable to the interviewee (49). This is a concern especially when conducting research with self-report data as it may interfere with the interpretation of average tendencies as well as individual differences (49). Sensitive topics that can lead to social desirability bias include personal income and earnings, reported health status, and as previously described, the use of certain pharmacologic therapies (50). Moreover, given the history and cultural background in the deep South, racial disparities may be amplified or unique compared to other parts of the country. However, a strength of this study is that it has consistently met recruitment goals, which has created a heterogeneous mixture by age, gender, and race. In general, many clinical research studies fail to meet or create recruitment goals to equally represent this distribution in funded research (51). In the current study, 58% of participants were women. This is consistent with the literature as it relates to women making up the majority of cases in cLBP (52) and reporting greater pain intensity (53) compared to men.

In conclusion, our findings suggest that NHWs living with cLBP may have more access to pharmacologic therapies, which may influence the ability to afford certain medications and obtain prescriptions compared to NHBs. Additional research is needed on age predictions for utilization of primary or tertiary care for cLBP discussed in this paper. Specifically, future research should seek to understand why younger adults with cLBP are more likely to have no provider for cLBP care. Future studies should continue to examine the influence of sociodemographic and SES factors to cLBP and compare with other types of chronic pain.

## Data Availability Statement

The raw data supporting the conclusions of this article will be made available by the authors, without undue reservation.

## Ethics Statement

The study protocol was approved by the Institutional Review Boards of the University of Alabama at Birmingham. All patients provided written informed consent.

## Author Contributions

KA-W, DD, DR, TLB, and AS are responsible for the main draft of the manuscript. BG conceived the study, oversees the trial design, and has overall responsibility of trial conduct. BG and DR provided substantial contributions to revising the manuscript. TQ oversees day-to-day operations and is instrumental in assisting with protocol development and study conduct. TWB reviewed the manuscript and provided critical feedback. BG and AS provided information on statistical analyses. All authors contributed to the article and approved the submitted version.

## Funding

Financial support for this research was provided by the National Institute on Minority Health and Health Disparities under award number R01MD010441 (BG) and by the National Institute on Aging under award number K02AG062498 (TWB). None of the authors have any conflict of interest to report.

## Conflict of Interest

The authors declare that the research was conducted in the absence of any commercial or financial relationships that could be construed as a potential conflict of interest.

## Publisher's Note

All claims expressed in this article are solely those of the authors and do not necessarily represent those of their affiliated organizations, or those of the publisher, the editors and the reviewers. Any product that may be evaluated in this article, or claim that may be made by its manufacturer, is not guaranteed or endorsed by the publisher.
